# Cannabidiol is a behavioral modulator in BTBR mouse model of idiopathic autism

**DOI:** 10.3389/fnins.2024.1359810

**Published:** 2024-05-09

**Authors:** Sarah H. Shrader, Nicholas Mellen, Jun Cai, Gregory N. Barnes, Zhao-Hui Song

**Affiliations:** ^1^Department of Pharmacology and Toxicology, University of Louisville School of Medicine, Louisville, KY, United States; ^2^Departments of Neurology and Autism Center, Pediatric Research Institute, University of Louisville School of Medicine, Louisville, KY, United States; ^3^Department of Pediatrics, Pediatric Research Institute, University of Louisville School of Medicine, Louisville, KY, United States

**Keywords:** cannabidiol, autism spectrum disorders, repetitive behaviors, social deficits, behavioral assays

## Abstract

**Introduction:**

The prevalence of Autism Spectrum Disorder (ASD) has drastically risen over the last two decades and is currently estimated to affect 1 in 36 children in the U.S., according to the center for disease control (CDC). This heterogenous neurodevelopmental disorder is characterized by impaired social interactions, communication deficits, and repetitive behaviors plus restricted interest. Autistic individuals also commonly present with a myriad of comorbidities, such as attention deficit hyperactivity disorder, anxiety, and seizures. To date, a pharmacological intervention for the treatment of core autistic symptoms has not been identified. Cannabidiol (CBD), the major nonpsychoactive constituent of *Cannabis sativa*, is suggested to have multiple therapeutic applications, but its effect(s) on idiopathic autism is unknown. We hypothesized that CBD will effectively attenuate the autism-like behaviors and autism-associated comorbid behaviors in BTBR T^+^Itpr3^tf^/J (BTBR) mice, an established mouse model of idiopathic ASD.

**Methods:**

Male BTBR mice were injected intraperitoneally with either vehicle, 20 mg/kg CBD or 50 mg/kg CBD daily for two weeks beginning at postnatal day 21 ± 3. On the final treatment day, a battery of behavioral assays were used to evaluate the effects of CBD on the BTBR mice, as compared to age-matched, vehicle-treated C57BL/6 J mice.

**Results:**

High dose (50 mg/kg) CBD treatment attenuated the elevated repetitive self-grooming behavior and hyperlocomotion in BTBR mice. The social deficits exhibited by the control BTBR mice were rescued by the 20 mg/kg CBD treatment.

**Discussion:**

Our data indicate that different doses for CBD are needed for treating specific ASD-like behaviors. Together, our results suggest that CBD may be an effective drug to ameliorate repetitive/restricted behaviors, social deficits, and autism-associated hyperactivity.

## 1 Introduction

Autism spectrum disorder (ASD) is a heterogenous group of neurodevelopmental disorders, clinically characterized by three core symptoms: impaired social interactions, social communication deficits, and repetitive behaviors with restricted interests (Park et al., 2016). With prevalence drastically rising over the last two decades to an estimated 1 in 36 children, ASD has become one of the most common neurodevelopmental disorders (Maenner et al., 2023). The molecular mechanisms underlying ASD pathogenesis remain elusive. The majority of cases are idiopathic, with only ~20–30% attributable to rare genetic variants including *de novo* mutations, copy number variants and monogenic disorders, such as Fragile X Syndrome, Tuberous Sclerosis Complex, and Rett Syndrome (Miles, 2011; Persico and Napolioni, 2013; Liu and Takumi, 2014). Based on twin and familial epidemiological studies, the etiology of ASD is thought to involve a complex interaction between diverse genetic, epigenetic and environmental risk factors (Hallmayer et al., 2011; Colvert et al., 2015; Deng et al., 2015; Tick et al., 2016).

Currently, no genetic, neuroimaging, or electrophysiological tests exists to definitively diagnose patients. Instead, ASD is clinically diagnosed by evaluation and characterization of behavioral, social and cognitive patterns, with a broad range and severity of symptoms unique to each patient (Sharma et al., 2018; Shulman et al., 2020). Onset of ASD typically occurs in early childhood, with most core symptoms appearing between 12 and 24 months of age (Zwaigenbaum et al., 2015). Physicians and researchers place an emphasis on the importance of early diagnosis, as considerable evidence supports the benefit of early intervention on long-term patient outcome (Dawson et al., 2010; Zwaigenbaum et al., 2015; Vivanti et al., 2016). In addition, ASD is often accompanied by several comorbidities, including attention deficit hyperactivity disorder (ADHD) and anxiety disorder, which introduces complexity in making diagnoses and establishing individualized treatment plans.

There is a significant unmet need for the development of therapeutic interventions for ASD (Masi et al., 2017). Standard of care at present relies heavily on behavioral therapy, which has varying success among this diverse patient population. Currently, risperidone and aripiprazole are the only two FDA-approved drugs for the treatment of autism-associated irritability and aggression (Eissa et al., 2018; Lamy et al., 2020). However, a pharmacological intervention for the treatment of core autistic symptoms has yet to be successfully developed.

*Cannabis sativa* has garnered recent attention for its potential therapeutic applications and is demonstrated to have analgesic, antiemetic, anticonvulsant, neuroprotective, anti-inflammation and antitumor properties (Atakan, 2012; McAllister et al., 2015; Alexander, 2016; Rosenberg et al., 2017). In particular, cannabidiol (CBD), the primary nonpsychoactive component of *Cannabis*, has been approved by the US FDA for the treatment of seizures associated with Lennox–Gastaut syndrome, Dravet syndrome, and tuberous sclerosis complex (Epidiolex, 2022). Case reports and pilot clinical trials have suggested that the cannabinoid may also be effective in targeting core autistic symptoms (Pedrazzi et al., 2022). However, the efficacy of purified CBD in idiopathic ASD has not been thoroughly investigated in preclinical or clinical studies.

BTBR T^+^Itpr3^tf^/J (BTBR) mice are an inbred mouse strain carrying the mutations *a^t^* (nonagouti; black and tan), Itpr3^tf^ (inositol 1,4,5-triphosphate receptor 3; tufted), and *T* (brachyury).^1^ BTBR mice have become an established model of idiopathic ASD, in part due to known ASD-associated genetic mutations and their strong behavioral face validity (McFarlane et al., 2008; Ellegood and Crawley, 2015; Meyza and Blanchard, 2017). As compared to C57BL6/J (B6) mice controls, BTBR mice exhibit reduced social interaction (Bolivar et al., 2007; Moy et al., 2007; Yang et al., 2007; McFarlane et al., 2008), increased repetitive self-grooming (McFarlane et al., 2008; Silverman et al., 2010; Pearson et al., 2011; Amodeo et al., 2012), and increased locomotor activity (McFarlane et al., 2008; Molenhuis et al., 2014; Chao et al., 2018; Faraji et al., 2018).

CBD has previously been found to attenuate social deficits in *Scn1a ^+/−^* mice, which is a mono-genetic mouse model of Dravet syndrome that exhibits autism-like behaviors (Kaplan et al., 2017; Patra et al., 2020). Recently, a single acute 10 mg/kg dose of CBD was shown to improve social interactions in adult BTBR mice (Ferreira et al., 2023). However, to our knowledge, the chronic effects of CBD, which are translationally important for mimicking potential clinical applications, have never been investigated in the BTBR strain. In addition, the effects of CBD on other autism-like behaviors, such as repetitive behavior and hyperactivity, have never been studied in any animal models of autism.

The current study tested the hypothesis that CBD administered chronically in the immediate post-weaning period can rescue both core behavioral deficits and autism-associated comorbid symptoms in a mouse model of idiopathic ASD. Behavioral assays were implemented to examine repetitive self-grooming, sociability, and locomotor activity in BTBR mice, following daily intraperitoneal (I.P.) treatment of vehicle or CBD for two weeks.

## 2 Materials and methods

### 2.1 Materials

Cannabidiol was purchased from Cayman Chemical (Ann Arbor, MI). Ethanol and Tween 20 were purchased from Sigma Aldrich (St Louis, MO). 1 mL syringes and 27-gauge needles were purchased from VWR (Radnor, PA).

### 2.2 Animal maintenance and housing

This study was conducted according to a protocol approved by the University of Louisville Institutional Animal Care and Use Committee (IACUC protocol number 19501) and NIH guidelines. Subjects were offspring of C57BL/6 J (B6) and BTBR T^+^Itpr3^tf^/J (BTBR) breeding pairs obtained from Jackson Laboratory (Bar Harbor, ME). Animals were bred and housed in clean, federally regulated and AAALAC-accredited facilities operated by the University of Louisville School of Medicine Department of Animal Care. Subjects were male B6 mice and BTBR mice between the ages of 3 weeks and 6 weeks of age. Mice were weaned at postnatal day (pnd) 21 and no more than five littermates were housed per cage (33 cm long x 19 cm wide x 15 cm high) with free access to food and water, in a humidity (30%) and temperature (22 ± 1°C) controlled room with a 12 h light/dark cycle (lights on at 0600 h). All animals utilized in this project were monitored daily for evidence of discomfort, distress, pain, or injury.

### 2.3 Animal dosing

This study investigated the effects of CBD on the behaviors of male BTBR mice. CBD was dissolved in 5% ethanol, 5% Tween 20 and PBS (137 mM NaCl, 2.7 mM KCl, 10 mM Na_2_HPO_4_, 1.8 mM KH_2_PO_4_). Weaned juvenile BTBR mice were administered daily via I.P. injection either vehicle, 20 mg/kg CBD or 50 mg/kg CBD treatment for two weeks, beginning at pnd21 ± 3 days. Following injection on the final treatment day (i.e., pnd34 ± 3), mice were subject to a battery of behavioral testing as outlined below. Male age-matched vehicle-treated B6 mice were used as controls. *N* = 10–15 mice/dosing group. Experiments were performed in 3 cohorts (*N* = 3–5 mice per cohort) with same experimental conditions for each cohort. In addition, for each cohort, all the experimental groups are included to avoid the impact of any possible variations of experimental conditions between cohorts. Furthermore, data are compiled from all the cohorts to minimize experimental variations (if any) between different cohorts.

### 2.4 Behavioral room set-up

All behavioral testing was conducted in an experimental room in the same building but separate from the animal care facilities. All experiments were conducted during the light cycle (Yang et al., 2008) between 0900 h and 1800 h. For each subject, the battery of behavioral assays was conducted within a single day. Following I.P. injection of drug or vehicle on final day of treatment, animals were brought in their home cages to the experimental room to acclimate for 30 min to 1 h prior to testing. The room was kept quiet during the entire time animals were present. The order of testing was chosen in order to minimize stress impact on the animal, starting with the least stressful test and ending with the most stressful. Thus, the testing sequence began with the repetitive self-grooming assay, followed by the open field assay and concluding with the three-chamber sociability assay. Subjects were given a minimum of 45 min resting period in their home cage between each assay. Behavioral apparatuses were thoroughly cleaned with 70% ethanol and deionized water between test subjects.

### 2.5 Machine vision analysis of behavior

All behavioral recordings were carried out under red light in a quiet, dark, temperature-controlled room dedicated to this purpose. Recordings were carried out in a sound-proofed enclosure built using modular aluminum elements (McMaster-Carr, Aurora OH) that support experimental enclosures made of Plexiglas, to standard dimensions (Crawley, 2004; Moy et al., 2004; Yang et al., 2008). Image acquisition software has been developed in-house, using the LabView programming environment (National Instruments, Austin, TX), running on a customized workstation (Windows 10, 32 GB RAM). Behavior was recorded by 2 cameras (Basler ace acA640-300gm GigE, Mono, 6 mm UC Series Lens; Edmund Optics) providing top- and side-views of enclosures to simultaneously record ongoing behaviors (30 Hz). Behavior was analyzed using in-house software developed in LabView. Background subtraction and machine-vision utilities were used to track the mouse’s movements. In-house software was benchmarked and validated against a commercially available package (Smart 3.0, Harvard Apparatus, Boston, MA) with similar results. Total path length, time spent in different parts of the enclosure, rearing and jumping were all automatically extracted. In addition, video segments corresponding to pauses in locomotion (based on minimum pause duration, and maximum movement during a pause) were extracted for subsequent inspection and manual scoring (grooming, social interactions, sniffing, object exploration). Fur color markings distinguished B6 mice (dark brown) from BTBR mice (dark brown with tan ventral patch), and prevented observers from being fully blind to strain during manual scoring of behaviors. All data were exported to excel (Microsoft, Redmond WA), and summary statistics for each experiment, and summaries of summaries were all generated using macros within Excel. More details can be found in Supplementary Figure S1.

### 2.6 Repetitive self-grooming assay

The repetitive self-grooming assay used to measure restricted interest/repetitive behavior was carried out following a previously published protocol (Yu et al., 2020). Briefly, a single mouse subject was placed in a clean, empty cage with 1 cm of bedding, and allowed to freely explore. The mouse’s behavior and movements were recorded for 10 min. From the video recordings, cumulative time spent self-grooming was scored by the researcher using automated image capture. Time spent performing different behaviors (rearing, grooming, etc.) was manually scored by the observer using automated image capture. Spontaneous self-grooming behavior included paw/leg licking, head washing, genital/tail grooming and body grooming. Fresh, clean cages were used for each subject. Subjects were returned to their home cages following testing.

### 2.7 Three-chamber sociability assay

The three-chamber assay evaluated the sociability phenotype of B6 and BTBR mice with or without CBD treatment following a published protocol (Yang et al., 2011). The three-chamber apparatus was a rectangular Plexiglas box with each chamber measuring 48 cm long x 19 cm wide × 25.5 cm high. Plexiglass walls (48 cm long) dividing the chambers contained 18 cm wide × 25.5 cm high openings (i.e., “doorways”) to allow access between the center and side chambers. Briefly, the subject mouse was given a 10 min habituation period in the middle chamber with side chambers blocked off. For Phase A, the side chambers were then opened so that the mouse was given free access to the entire arena and its movements were recorded for 10 min. For Phase B, the subject mouse was then briefly confined to the center chamber. A socially unfamiliar, age- and sex-matched B6 mouse (novel mouse) was placed in an inverted wire cup in the center of one the side chambers, while an empty inverted wire cup (novel object) was placed in the center of other side chamber. An upright beaker was placed on top of each inverted wire cup to prevent the subject from climbing onto the top of the wire cups. The side doors were then opened, the subject mouse was given free access to the entire arena and its movements were recorded for 10 min. The side chamber placement of the novel mouse and novel object was alternated between test mice, and a lack of innate side preference was confirmed during the 10 min habituation period to the empty arena. The time spent in each chamber was measured via the in-house Machine Vision tracking system and subject’s behavior was manually scored.

### 2.8 Open field assay

The open field assay was subsequently used to determine whether CBD had an effect on exploratory locomotor activity and anxiety-like behavior. The open field assay was conducted in a square white plexiglass chamber (60 cm long x 60 cm wide x 26 cm high) and followed a previously published protocol (Seibenhener and Wooten, 2015). Individual subjects were placed in the center of the open field chamber and allowed to freely explore. Mouse movements and behavior were recorded for 10 min. Distance traveled was measured using the in-house Machine Vision tracking system. Time spent in the center and edges of the chamber were recorded to assess anxiety-related behavior.

### 2.9 Statistical analyses

Data were plotted using GraphPad Prism 9 Statistical Software (San Diego, CA) and presented as mean ± SEM. Data from the self-grooming and open field assays were analyzed by one-way analysis of variances (ANOVAs) to compare groups. Bonferroni’s multiple comparisons post-hoc tests were performed when appropriate. Data from the three-chamber sociability assay were analyzed by multiple unpaired t-tests comparing novel mouse vs. novel object chamber times within groups. Statistical significance was set at *p* < 0.05.

## 3 Results

### 3.1 CBD attenuates repetitive behavior in BTBR mice

**Figure 1:**
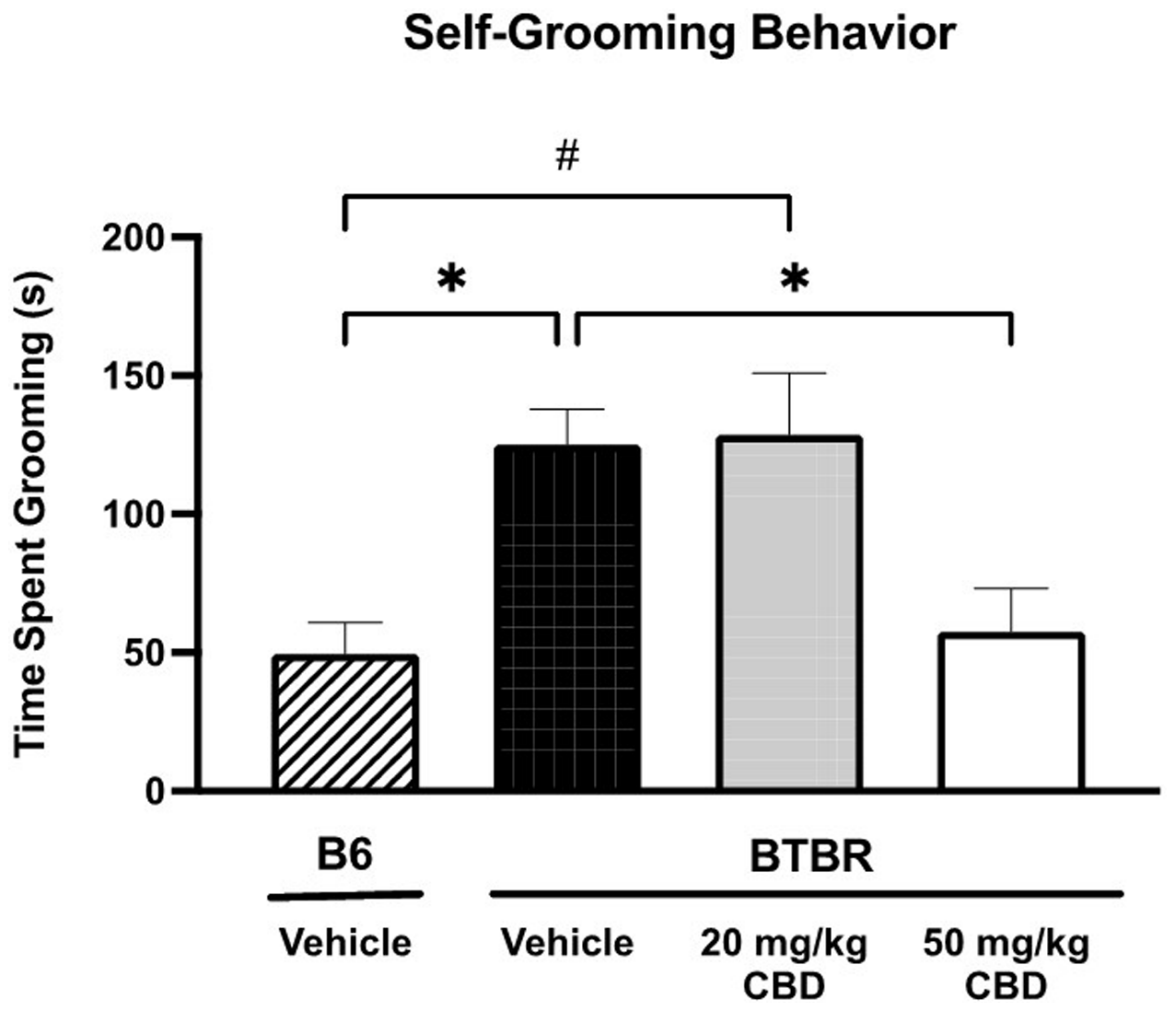
Repetitive behaviors in BTBR mice are attenuated by high dose CBD in self-grooming assay. Vehicle-treated BTBR mice exhibited increased grooming time compared to vehicle-treated B6 controls (*p* = 0.0161). Low dose CBD (i.e., 20 mg/kg) treatment had no significant effect on repetitive grooming behavior in BTBR mice, as self-grooming time was similar to vehicle-treated BTBR mice (*p* > 0.05) and significantly higher than vehicle-treated B6 mice (*p* = 0.0147). BTBR mice dosed with 50 mg/kg CBD showed significantly reduced grooming times compared to the vehicle-treated BTBR mice (*p* = 0.0168). Grooming time did not significantly differ between the 50 mg/kg CBD-treated BTBR mice and vehicle-treated B6 mice (*p* > 0.05). Data are presented as mean ± SEM. *N* = 10–15 mice/group. *p < 0.05 compared to vehicle-treated BTBR mice. #*p* < 0.05 compared to vehicle-treated B6 mice.

The repetitive self-grooming assay was used to measure restricted interest/repetitive behavior phenotype of B6 and BTBR mice (Figure 1). Vehicle-treated BTBR mice spent significantly more time self-grooming (mean = 125.20 ± 12.59 s; *N* = 14) compared to vehicle-treated B6 mice (mean = 49.39 ± 11.34 s; *N* = 10) (*p* = 0.0161). Chronic 20 mg/kg CBD dosing in BTBR mice did not alter self-grooming behavior (mean = 128.56 ± 22.11 s; *N* = 12). In the 20 mg/kg CBD-treated BTBR mice, time spent grooming was significantly elevated compared to vehicle-treated B6 mice (*p* = 0.0147), but not significantly different from vehicle-treated BTBR mice (*p* > 0.05). However, treatment with 50 mg/kg CBD in BTBR mice significantly attenuated the repetitive self-grooming behavior (mean = 57.51 ± 15.70 s; *N* = 15) compared to vehicle-treated BTB mice (*p* = 0.0084) and 20 mg/kg CBD-treated BTBR mice (*p* = 0.0158). Notably, the reduced levels of grooming time seen in these 50 mg/kg CBD treated mice were comparable to that of the vehicle-treated B6 mice (*p* > 0.05).

### 3.2 CBD rescues social deficits in BTBR mice

**Figure 2:**
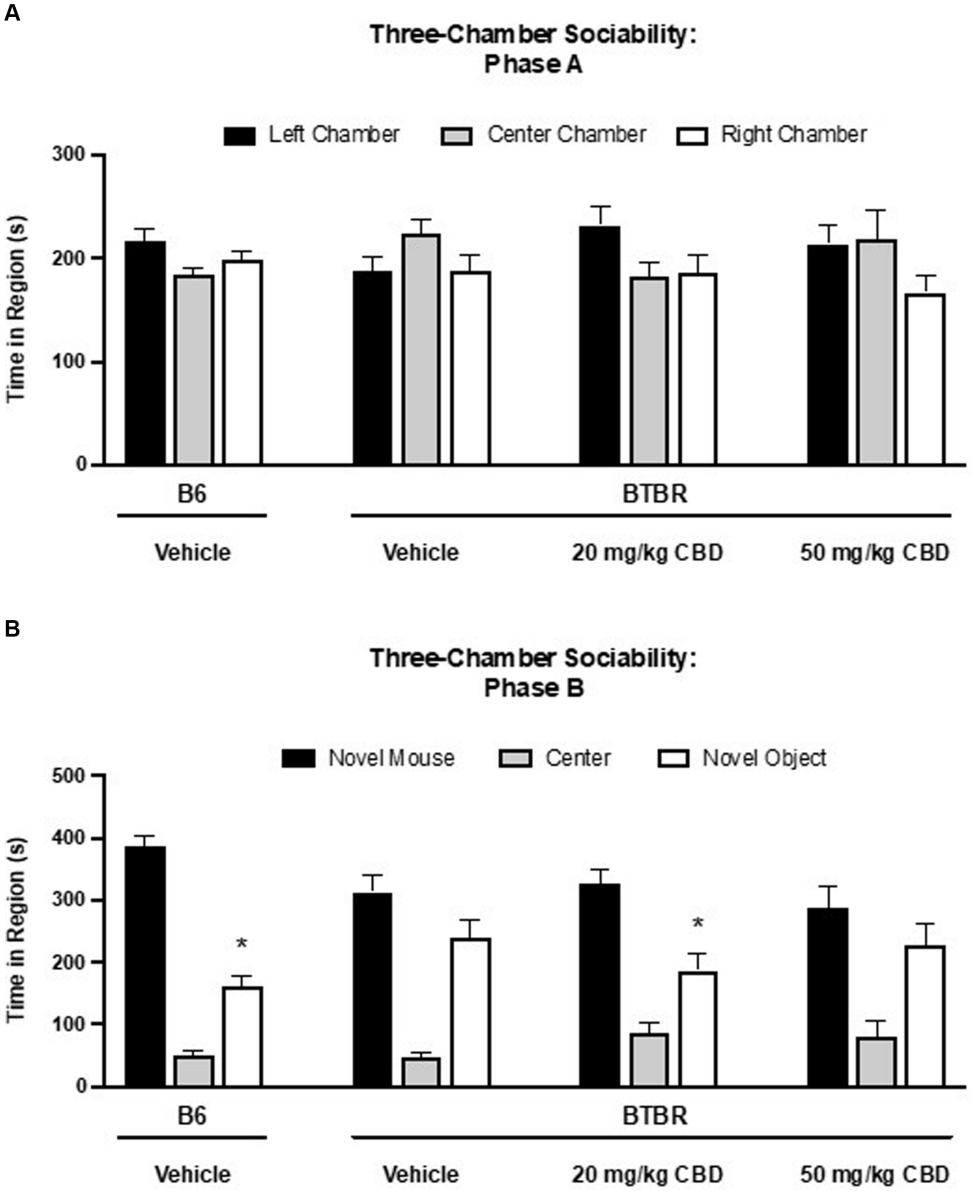
Sociability deficits in BTBR mice are rescued by low dose CBD in 3-chamber sociability assay. **(A)** Time spent in three chambers prior to the introduction of novel mouse and novel subject. There are no difference among the time spent in the three chambers. **(B)** Time spent in three chambers after the introduction of novel mouse and novel subject. Vehicle-treated B6 mice spent more time in the chamber containing the novel mouse than the chamber containing the novel object (*p* < 0.000001). BTBR mice treated with vehicle demonstrated no social preference for the novel mouse over the novel object (*p* > 0.05), indicating sociability deficits. A social preference for the chamber with the novel mouse vs. the novel object was observed in BTBR mice following 20 mg/kg CBD treatment (*p* = 0.000576). BTBR mice dosed with 50 mg/kg CBD displayed asocial behavior with no difference in time spent in the novel mouse vs. the novel object chambers (*p* > 0.05). Data are presented as mean ± SEM. *N* = 12–15 mice/group. *p < 0.05 within group comparison of novel mouse vs. novel object chamber time.

The three-chamber assay was used to assess the sociability of BTBR and B6 mice (Figure 2). In phase A (Figure 2A), during which time the mice explored the entire empty three chamber arena, there was no statistically significant difference in the time spent in the left, center and right chambers for any of the four dosing groups. In phase B (Figure 2B), social preference was assessed by the amount of time spent in the chambers with the novel (socially unfamiliar) mouse and the novel object. Vehicle-treated B6 control mice spent more time in the novel mouse chamber compared to the novel object chamber (novel mouse mean = 386.27 ± 17.49 s; novel object mean = 162.59 ± 15.03 s; SE of difference = 23.05 s; *N* = 12) (*p* < 0.000001). In contrast, vehicle-treated BTBR mice displayed diminished sociability, showing no significant preference between the novel mouse and novel object (novel mouse mean = 312.73 ± 28.88 s; novel object mean = 238.73 ± 30.35 s; SE of difference = 41.89 s; *N* = 14) (*p* > 0.05). 20 mg/kg CBD treatment rescued the social deficits observed BTBR mice as time spent in the novel mouse chamber was significantly greater than time in the novel object chamber (novel mouse mean = 326.64 ± 22.43 s; novel object mean = 186.94 ± 27.18 s; SE of difference = 35.24 s; *N* = 13) (*p* = 0.000576). This rescuing effect was specific to the 20 mg/kg dosage. No significant changes in the asocial behavior of BTBR mice were observed following 50 mg/kg CBD treatment (novel mouse mean = 289.17 ± 32.20 s; novel object mean = 227.30 ± 34.19 s; SE of difference = 46.96 s; *N* = 15) (*p* > 0.05).

### 3.3 CBD reduces hyperlocomotion, but has no effect on anxiety-like behavior in BTBR mice

**Figure 3:**
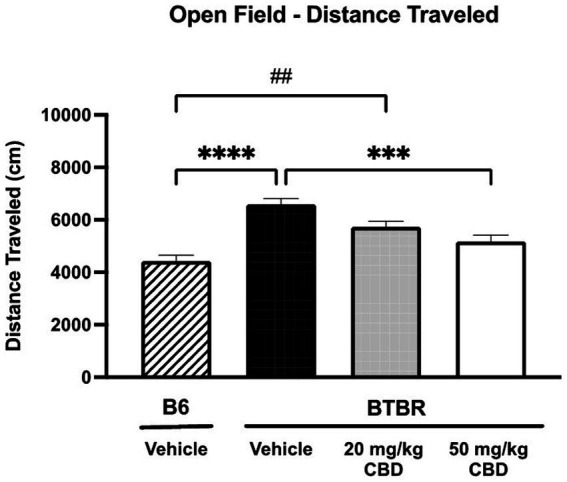
Hyperlocomotion in BTBR mice is reduced by CBD in open field assay. Distance traveled by vehicle-treated BTBR mice was significantly higher than that of B6 controls (*p* < 0.0001), suggesting hyperactivity. BTBR mice dosed with 20 mg/kg CBD traveled distances that were similar to vehicle-treated BTBR mice (*p* > 0.05) and significantly greater than vehicle-treated B6 mice (*p* = 0.0023). 50 mg/kg CBD treatment in BTBR mice significantly reduced locomotor activity compared to vehicle-treated BTBR mice (*p* = 0.0003). There was no significant difference in distance traveled by 50 mg/kg CBD-treated BTBR mice and vehicle-treated B6 mice (*p* > 0.05). Data are presented as mean ± SEM. *N* = 11–15 mice/group. ***p < 0.001 and ****p < 0.0001 compared to vehicle-treated BTBR mice. ##*p* < 0.01 compared to vehicle-treated B6 mice.

**Figure 4:**
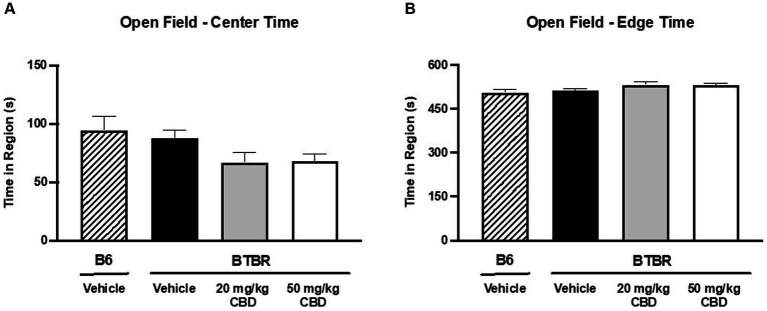
CBD has no effect on anxiety-like behavior in BTBR mice in open field assay. **(A)** When comparing time spent in the center of the open field, no significant difference was observed between vehicle-treated BTBR and vehicle-treated B6 mice (*p* > 0.05), indicating comparable levels of anxiety. Furthermore, CBD treatment in BTBR mice did not have an effect on center region time for either the 20 mg/kg dose (*p* > 0.05 compared to BTBR vehicle and B6 vehicle) or the 50 mg/kg dose (*p* > 0.05 compared to BTBR vehicle and B6 vehicle). **(B)** Congruently, time spent in the edge of the open field was similar across all four groups (*p* > 0.05). Data are presented as mean ± SEM. *N* = 11–15 mice/group.

Because hyperactivity and anxiety are two common ASD co-morbidities (Lai et al., 2019), exploratory locomotor activity (Figure 3) and anxiety-like behavior (Figure 4) was tested in BTBR and B6 mice using the open field assay. As shown in Figure 3, the distance traveled by vehicle-treated BTBR mice (mean = 6590.64 ± 221.38 cm; *N* = 15) was significantly higher than that of the vehicle-treated B6 mice (mean = 4426.15 ± 425.90 cm; *N* = 15) (*p* < 0.0001). The 20 mg/kg CBD treatment did not appear to have a significant effect on the hyperlocomotor activity, as the distance traveled (mean = 5735.65 ± 213.99 cm; *N* = 11) was similar to that of vehicle-treated BTBR mice (*p* > 0.05) and still significantly higher than that of vehicle-treated B6 mice (*p* = 0.0023). However, the distance traveled by BTBR mice was significantly reduced following the 50 mg/kg CBD treatment (mean = 5172.42 ± 251.38 cm; *N* = 15) compared to vehicle-treated BTBR mice (*p* = 0.0003). Due to this decrease, locomotor activity levels were similar in the 50 mg/kg CBD-treated BTBR mice and the vehicle-treated B6 mice (*p* > 0.05).

Anxiety-like behavior was assessed by measuring the duration of time spent near the center of the open field (Figure 4A) vs. the edge (Figure 4B) over the 10 min testing period. Greater time near the perimeter indicated higher levels of anxiety-like behavior (Seibenhener and Wooten, 2015). There were no significant differences in time spent in the center of the field or in the edge of the field when comparing B6 controls (center mean = 94.45 ± 11.79 s; edge mean = 505.35 ± 11.79 s; *N* = 15) and vehicle-treated BTBR mice (center mean = 87.79 ± 6.71 s; edge mean = 512.21 ± 6.69 s; *N* = 15) (*p* > 0.05). Furthermore, CBD treatment had no effect on anxiety-like behavior at either the 20 mg/kg dosage (center mean = 66.81 ± 8.75 s; edge mean = 533.08 ± 8.74 s; *N* = 11) (*p* > 0.05 compared to BTBR vehicle and B6 vehicle) or the 50 mg/kg dosage (center mean = 67.90 ± 6.21 s; edge mean = 532.10 ± 6.20 s; *N* = 15) (*p* > 0.05 compared to BTBR vehicle and B6 vehicle).

## 4 Discussion

Autism is such a uniquely human condition that developing a well-validated animal model for translational research presents many challenges. Monogenic models of ASD, such as Fragile X Syndrome (FXS) *Fmr1* mutant mice, Tuberous Sclerosis Complex (TSC) *TSC1* or *TSC2* mutant mice and Rett Syndrome *Mecp2* mutant mice, provide good construct validity for exploring potential therapeutic avenues. However, autistic individuals with these single-gene disorders compose only a small subset of the overall autism population, roughly 5–10% (Miles, 2011; Persico and Napolioni, 2013; Liu and Takumi, 2014). The vast majority of cases are idiopathic in nature (Miles, 2011; Persico and Napolioni, 2013; Liu and Takumi, 2014).

In this study, we investigated the effects of CBD on ASD-associated behavioral phenotypes as recapitulated in the BTBR mouse model of idiopathic autism. This particular animal model of ASD has strong behavioral face validity for the aberrant behaviors examined here, which are thought to be regulated by polygenic mutations also seen in clinical idiopathic ASD cases (Meyza and Blanchard, 2017). Furthermore, this inbred mouse strain has numerous genomic, proteomic, neurophysiologic, anatomic, and synaptic protein changes which are similar to observations in both monogenic ASD mouse models and ASD patients (Meyza and Blanchard, 2017).

The time of initiation and duration of treatment for ASD is a critical factor in long-term patient outcome (Zwaigenbaum et al., 2015). Studies have shown that early and continuous intervention during childhood can lead to improved developmental trajectories in individuals with ASD (Dawson et al., 2010; Virués-Ortega, 2010; Zwaigenbaum et al., 2015; Vivanti et al., 2016; Linstead et al., 2017; Huang et al., 2020). As such, we chose to begin chronic CBD treatment around the time of weaning on pnd 21 (± 3 days). Although fraught with difficulties, developmental comparisons between species have led researchers to correlate this mouse age to roughly 1–2 human years of age (Dutta and Sengupta, 2016), which is when clinical ASD can be reliably diagnosed (Zwaigenbaum et al., 2015). A two-week dosing regimen was used in order to mimic chronic treatment from time of diagnosis through childhood (pre-puberty). Following the two-week daily I.P. treatment with either vehicle, 20 mg/kg or 50 mg/kg CBD doses, we investigated the effect of CBD using a battery of behavioral assays that measured repetitive self-grooming and sociability, as well as autism-associated hyperactivity and anxiety. In the future, it would be very interesting to study whether the effects of CBD persist after stopping the drug and how long will the effects last.

Excessive barbering and self-grooming, a common characteristic of BTBR mice, is considered similar to the repetitive behaviors seen in autistic individuals. In line with prior studies (Silverman et al., 2010; Amodeo et al., 2016; Ansari et al., 2017; Cai et al., 2017), vehicle-treated BTBR mice spent significantly more time self-grooming compared to vehicle-treated B6 mice. We further discovered that this increased repetitive behavior was significantly reduced in BTBR mice receiving high dose (50 mg/kg), but not the low dose (20 mg/kg) CBD treatment.

A recent meta-analysis found that current pharmacological treatments for restricted, repetitive behaviors (RRBs) in ASD provide only mild benefits at best (Zhou et al., 2021). Of the various classes of drugs analyzed, antipsychotics, specifically risperidone and aripiprazole, demonstrated the greatest positive effects (Zhou et al., 2021). Clinicians, however, need to weigh the minimal benefits against the significant adverse effects associated with antipsychotics, such as metabolic weight gain and fatigue (Muench and Hamer, 2010). The minimal effects of antipsychotics on repetitive behavior is also consistent with a previous study in a mono-genetic model of ASD showing that genetic rescue of SHANK2 during early neonatal period improved social behavior, but did not restore the malfunctioned brain circuit responsible for repetitive behaviors (Eltokhi et al., 2021). This indicates that repetitive behavior may be hard-wired and not showing any plasticity to the point that it cannot be rescued after genetic modification. In contrast, the current pre-clinical study in BTBR mice suggests that an appropriate dose of CBD has the potential to alleviate the repetitive behavior in ASD patients, if treated right after ASD diagnosis at 1–2 years of age and prior to puberty, before the repetitive behaviors are being hard-wired.

Risperidone and aripiprazole are currently the only two FDA-approved drugs for the treatment of autism-associated irritability and aggression. These atypical (second generation) antipsychotics have been shown to mildly improve stereotypy and repetitive behaviors (McDougle et al., 1998; McCracken et al., 2002; Shea et al., 2004; Marcus et al., 2009; Owen et al., 2009). The mechanism of action of risperidone is thought to involve antagonism of the dopamine type 2 (D2) receptors and serotonin type 2A (5-HT_2A_) receptors; whereas aripiprazole is posited to act via partial agonism/antagonism of D2 and 5-HT_1A_ receptors, along with 5-HT_2A_ receptor antagonism (Posey et al., 2008; de Bartolomeis et al., 2015).

The findings of the present study suggest that cannabidiol (CBD), when administered at an appropriate dose (50 mg/kg), could serve as a potential alternative therapeutic agent for the restricted, repetitive behaviors observed in patients with autism spectrum disorder (ASD). However, the precise mechanisms underlying CBD-induced reduction of repetitive behavior remain to be fully elucidated. While sedation has been reported as a potential side effect of CBD, it is more plausible that CBD influences the neural circuitry associated with repetitive behaviors.

The neural circuits underlying repetitive behaviors are exceedingly intricate, primarily involving the cortico-basal ganglia-thalamic pathway (Kim et al., 2016). This pathway comprises both direct pathways, characterized by dopamine D1 receptor expression, and indirect pathways, characterized by dopamine D2 receptor expression. The direct pathway facilitates movement, while the indirect pathway inhibits it (Kim et al., 2016). Notably, BTBR mice demonstrate significant reductions in both pre- and postsynaptic dopamine D2 receptor function (Squillace et al., 2014), which may explain this behavioral phenotype.

Seeman (2016) demonstrated that CBD displayed partial agonist activity *in vitro* at dopamine D2 receptors in rat striatal tissues. We more recently found CBD to act *in vivo* as a partial agonist on dopamine D2-like receptors in the nematode *Caenorhabditis elegans (C. elegans)* (Shrader et al., 2020). This suggests that CBD may compensate for the reduction in both pre- and postsynaptic dopamine D2 receptor function, thereby modulating the dopamine indirect pathway implicated in the repetitive behaviors.

The three-chamber sociability test is commonly used to measure the presence or absence of social preference by comparing the time spent in the chamber with an age-matched, sex-matched novel mouse to the time spent in the chamber with an object (an empty cup). Numerous studies have demonstrated that BTBR mice exhibit reduced social interaction (Bolivar et al., 2007; Yang et al., 2007; McFarlane et al., 2008). In this study, we were able to confirm a lower level of sociability in vehicle-treated BTBR mice, as compared to their B6 counterparts. In addition, the 20 mg/kg CBD treatment attenuated the social deficit, while no significant effect was observed with the 50 mg/kg CBD treatment. These data suggest that an appropriate dose of CBD may have therapeutic potential to treat the social deficits of ASD patients. This effect on social behavior is supported by a very recent report that an acute single dose of 10 mg/kg CBD, but not 0.1 or 1 mg/kg CBD, was able to enhance social interaction preference in adult BTBR mice (Ferreira et al., 2023). While the specific mechanisms of action by which CBD elicits its effects on sociability is currently not clear, it is known that CBD has multiple molecular targets (Bisogno et al., 2001; Di Marzo and Piscitelli, 2015; Laun et al., 2019). It is possible that at 20 mg/kg, CBD acts on a certain target to improve sociability, whereas at 50 mg/kg, additional targets of CBD might be activated which counteract the beneficial effects of CBD on sociability.

The mesocorticolimbic (MCL) dopaminergic pathway, which consists of the ventral tegmental area, shell and core parts of the nucleus accumbens, and medial prefrontal cortex, is known to mediate social behavior in humans and mice (Nakamura et al., 2023). Previously, it has been reported that selective PPARγ agonists inhibit mesocorticolimbic dopamine activity and block neuropsychiatric symptoms (Jung et al., 2022). Since CBD is a known PPARγ agonist, it is possible that CBD may produce its sociability rescuing effects in BTBR mice by affecting the MCL dopaminergic pathway (Jung et al., 2022).

ADHD and anxiety disorder are two common ASD co-morbidities (Lai et al., 2019). The open field assay is frequently used to test exploratory locomotor activity and anxiety-like behavior in rodents. In the current study, vehicle-treated BTBR mice exhibited hyperlocomotor activity compared to B6 control mice. This finding is in line with numerous published studies (Molenhuis et al., 2014; Golubeva et al., 2017; Faraji et al., 2018; O’Connor et al., 2021). Notably, we further observed a significant reduction in hyperlocomotor activity in BTBR mice following chronic dosing of CBD. Prior research on anxiety-like behavior in BTBR mice using the open field test have yielded conflicting results, with some demonstrating increased anxiety-like behavior compared to B6 mice (Chao et al., 2018; Eissa et al., 2021) and others showing no difference (Golubeva et al., 2017; O’Connor et al., 2021). The age of the BTBR mice tested could in part play a factor in this discrepancy (Martin et al., 2014). In this study, time spent in the center of the field was similar between the vehicle-treated BTBR and B6 groups, suggesting comparable levels of anxiety in these strains. Moreover, neither dose of CBD had a significant effect on anxiety-like behavior in the BTBR mice. Together, these open field data suggest that CBD may be a potential treatment for the hyperactivity associated with ASD, but perhaps not effective in attenuating co-occurring anxiety.

In 2018, CBD (Epidiolex) became the first FDA-approved cannabis-derived drug. It is currently indicated for the treatment of seizures associated with Lennox–Gastaut syndrome, Dravet syndrome, and TSC (Epidiolex, 2022). Reports vary in terms of the comorbid prevalence of epilepsy in patients with ASD, but some studies have estimated close to 50% (Buckley and Holmes, 2016; Besag, 2018). Notably, some patients with Dravet Syndrome or TSC display autistic behaviors, and a portion of these individuals are co-diagnosed with ASD (Li et al., 2011; Henske et al., 2016; Strasser et al., 2018; Samanta, 2020).

The data presented in this study on the effect of CBD in an idiopathic ASD model are consistent with the previous preclinical findings related to Dravet syndrome. Kaplan et al. (2017) demonstrated in the *Scn1a ^+/−^* mouse model of Dravet syndrome that the autism-like social impairments seen in the three-chamber sociability test were improved following acute I.P. injection of CBD at doses of 10 mg/kg and 20 mg/kg. CBD was also able to attenuate seizures at high doses (100 mg/kg and 200 mg/kg) (Kaplan et al., 2017). More recently, Patra et al. (2020) found that twice daily subcutaneous 100 mg/kg CBD injections administered chronically for 4 weeks improved sociability and anxiety-like behavior in this same *Scn1a ^+/−^* mouse model. Therefore, both our data on BTBR mice and previous data from studies using animal models of Dravet syndrome demonstrate the potential of CBD for treating ASD.

The choice in this study to use only male mice was in part due to the male preponderance observed in clinical ASD, with an estimated male-to-female ratio of 3.8:1 (Maenner et al., 2023). Furthermore, female rodent estrus cycles has been shown to affect social behavior (Chari et al., 2020; Shanley et al., 2023) and anxiety-like behavior (Meziane et al., 2007; Lovick and Zangrossi, 2021), and thus, can add confounding factors that make interpretation of results difficult. Nevertheless, one aspect that warrants future investigation is the effects of CBD in female mice and the potential for sexual dimorphisms.

In the current study, CBD impacted repetitive behaviors, social deficits, and hyperactivity in the BTBR mice. We found that 20 mg/kg of CBD is effective for sociability, 50 mg/kg is effective for repetitive behavior and both doses are effective for hyperactivity in BTBR mice. These results are not entirely surprising since the FDA indication for Lennox Gastaut syndrome associated seizures is 20 mg/kg CBD Oil (Epidiolex) while the FDA indication for tuberous sclerosis associated seizures is 50 mg/kg (Epidiolex, 2022). Thus, human clinical trials have found variations in CBD dosing are required for control of various seizure types. Human clinical trials testing CBD for the treatment of ASD are currently ongoing.^2^ Data from pilot studies and case reports, though, are consistent with our preclinical findings and suggest that the CBD may be effective in alleviating both core and comorbid autistic symptoms (Barchel et al., 2018; Aran et al., 2019, 2021; Hacohen et al., 2022; Pedrazzi et al., 2022). The application of a tractable polygenic mouse model of idiopathic ASD such as BTBR mice is significant to answer many questions that have arisen in human clinical trials of CBD. In the future, to help design better clinical trials, more preclinical studies are warranted to determine the dependence of CBD efficacy on dose, sex of the animals, and age of the initiation of treatment.

## 5 Conclusion

Our understanding of ASD has been ever evolving since Grunya Efimovna Sukhareva’s initial observations of “infantile autism” in 1926 (Manouilenko and Bejerot, 2015; Posar and Visconti, 2017). Once considered a rare disorder with narrow diagnostic criteria, autism is now acknowledged to be a uniquely heterogenous condition characterized by broad genetic variability and a spectrum of symptomology. The core behavioral hallmarks – social communication deficits and repetitive behavior with restricted interests – can range from mild to severe, and be accompanied by a myriad of comorbidities such as ADHD and anxiety. There is a significant unmet need for the development of therapeutic interventions for ASD which may target the core autistic symptoms. This study demonstrates, for the first time, that repeated CBD dosing in juvenile BTBR mice during the first two weeks post-weaning (i.e., pnd21-34) can attenuate autism-related core and comorbid behaviors. Furthermore, our novel findings indicate that specific doses of CBD are needed to attenuate different ASD-associated behaviors: for example, 20 mg/kg CBD was effective for rescuing sociability deficits, whereas 50 mg/kg CBD was effective for reducing repetitive behaviors. Together, this study indicates the therapeutic efficacy of CBD in a preclinical model of idiopathic ASD, and suggests that the proper dosage of CBD administered chronically beginning at an early age may be clinically useful in targeting distinct core and comorbid symptoms in ASD patients.

## Data availability statement

The raw data supporting the conclusions of this article will be made available by the authors, without undue reservation.

## Ethics statement

The animal study was approved by IACUC of University of Louisville. The study was conducted in accordance with the local legislation and institutional requirements.

## Author contributions

SS: Conceptualization, Formal analysis, Investigation, Methodology, Validation, Visualization, Writing – original draft. NM: Methodology, Writing – review & editing. GB: Conceptualization, Funding acquisition, Project administration, Supervision, Writing – review & editing. Z-HS: Conceptualization, Funding acquisition, Methodology, Project administration, Supervision, Writing – review & editing. JC: Methodology, Writing – review & editing.

## Footnotes

^1^ http://jaxmice.jax.org/strain/002282.html

^2^ clinicaltrials.gov
