# Supplementary material for: Cannabidiol is a behavioral modulator in BTBR mouse model of idiopathic autism
Source: Front Neurosci. 2024 May 9;18:1359810. doi: 10.3389/fnins.2024.1359810 (PMC11112039; doi:10.3389/fnins.2024.1359810)
Supplement: Supplementary file 1 [file Table_1.DOCX]

Supplemental Figure 1: Machine vision extraction of mouse trajectory


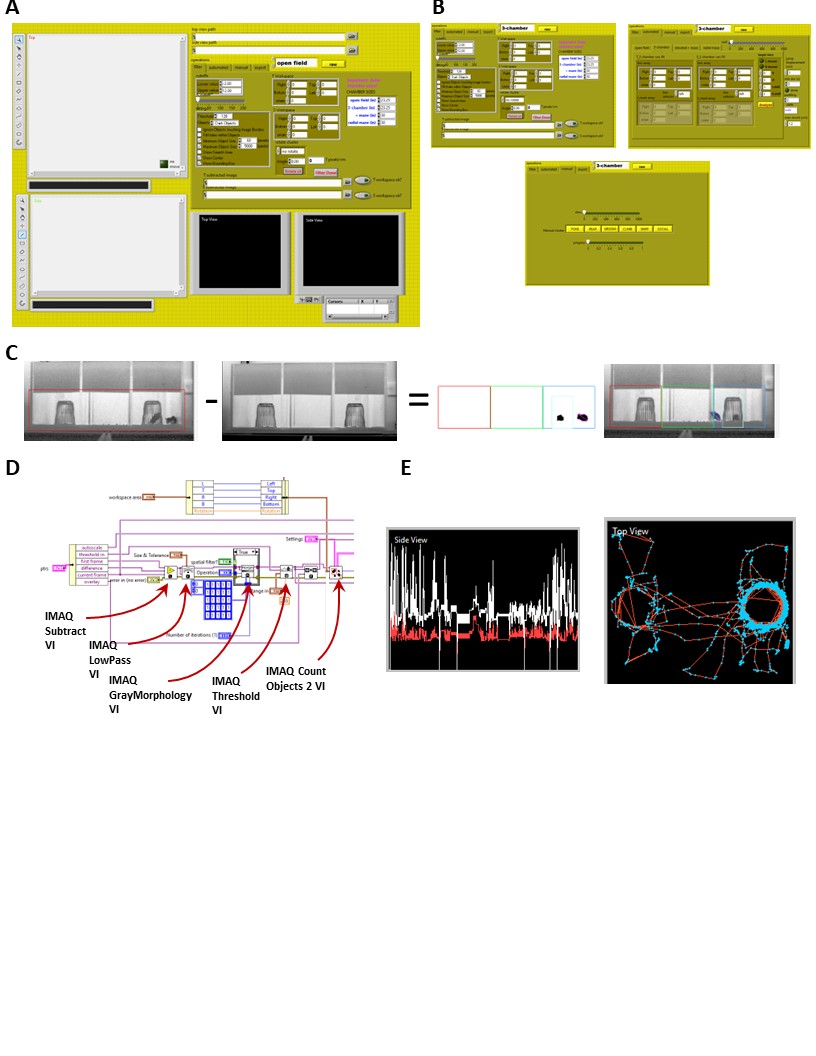


A. The GUI: White rectangles on the left display top and side views of the enclosure. Control panel in the center consists of 4 tabs. B. Tabs of Control Panel: The first filters the image to subtract the background and parametrize machine vision extraction of the target mouse. The second tab enables configuration of enclosure boundaries, definition of mask to exclude mice enclosed in cups from machine vision analysis, and specification of duration movement limits to select out image sequences when locomotion stops. The third tab allows the experimenter to classify non-locomotor behaviors as either poke, rear, groom, climb, sniff, or social interactions. The fourth tab enables export of video. C. Overview of machine vision approach to extracting mouse movement. Images obtained while the mouse is held in the enclosure (left) are subtracted from an image of the empty enclosure (second from left). Thresholding and spatial averaging allows for binarization of the image in which the mouse is shown in black on a white background, patency of tracking can be monitored in real time because while the mouse is tracked, it is outlined in red, and its main axis is shown in pink (second from right). Once tracking is optimized, behavior inside the enclosure can be monitored without filtering, with the target mouse outlined in red, and familiar and/or novel mice (enclosed in inverted cups) are masked out (light-blue rectangle). D. LabView code responsible for background subtraction, binarization and object tracking is shown, with labeling of relevant LabView-native subroutines. E. Output of mouse tracking from side camera (left) and overhead camera (right). Side camera data tracks highest point along main axis (white line), and center of mass (red line) discontinuities and unchanging values are due to periods when the target mouse was occluded by the mask. Overhead camera data tracks the location of the animal’s center of mass in each image (blue dots), with transition between images shown as red lines. All data are exported to excel for subsequent analysis.
